# Validation of the Impostor Phenomenon among Managers

**DOI:** 10.3389/fpsyg.2016.00821

**Published:** 2016-06-02

**Authors:** Sonja Rohrmann, Myriam N. Bechtoldt, Mona Leonhardt

**Affiliations:** ^1^Department of Psychology, Goethe University Frankfurt, FrankfurtGermany; ^2^Department of Management, Frankfurt School of Finance and Management, FrankfurtGermany

**Keywords:** impostor phenomenon, confirmatory factor analysis, construct validity, gender, strain

## Abstract

Following up on earlier investigations, the present research aims at validating the construct *impostor phenomenon* by taking other personality correlates into account and to examine whether the impostor phenomenon is a construct in its own right. In addition, gender effects as well as associations with dispositional working styles and strain are examined. In an online study we surveyed a sample of *N* = 242 individuals occupying leadership positions in different sectors. Confirmatory factor analyses provide empirical evidence for the discriminant validity of the impostor phenomenon. In accord with earlier studies we show that the impostor phenomenon is accompanied by higher levels of anxiety, dysphoric moods, emotional instability, a generally negative self-evaluation, and perfectionism. The study does not reveal any gender differences concerning the impostor phenomenon. With respect to working styles, persons with an impostor self-concept tend to show perfectionist as well as procrastinating behaviors. Moreover, they report being more stressed and strained by their work. In sum, the findings show that the impostor phenomenon constitutes a dysfunctional personality style. Practical implications are discussed.

## Introduction

In the 1970s the psychotherapists Clance and Imes (1978) noticed that some highly educated female individuals, objectively highly successful women, reported an extreme fear of failure. Instead of gaining self-confidence from their professional or academic success, they felt uncertain about it and attributed it to other factors than intelligence, for instance, personal charm, luck, or hard work. The significant discrepancy between these women’s self-views and their achievements inspired Clance and Imes (1978, p. 241) to coin the term “impostor phenomenon,”^1^ which they defined as “an internal experience of intellectual phoniness that appears to be particularly prevalent and intense among a select sample of high achieving women… despite outstanding academic and professional accomplishments, women who experience the impostor phenomenon persist in believing that they are really not bright and have fooled anyone who thinks otherwise”. Accordingly, the core characteristics of the impostor phenomenon have been described as (1) the sense of having fooled others into overestimating one’s ability, (2) the attribution of success to some other factor than intelligence or ability, and (3) the fear of being exposed as a fraud (Harvey and Katz, 1985, cf. Leary et al., 2000).

### Gender Differences

In the beginning Clance and Imes (1978) assumed that the impostor phenomenon affects particularly women and thus is gender-typical. This seems to be confirmed by investigations documenting that women tend toward lower self-evaluations than men (e.g., Bischof-Köhler, 2006). Moreover, research on the attribution of success has revealed that girls tend to attribute success above all to external factors, such as luck or the simplicity of a task, whereas boys tend to attribute achievements to themselves and their own abilities, i.e., internal factors (e.g., Bischof-Köhler, 2006). In all, the findings of these studies confirm the original assumption of Clance and Imes (1978) that the impostor phenomenon is a typically female pattern of experience. Subsequent investigations, however, yielded contradictory results regarding potential gender differences: King and Cooley (1995) confirmed the assumption of Clance and Imes; they found significantly higher impostor values in female than in male college students. The same was observed in a study among German students of psychology (Spinath, 2010), whereas Fried-Buchalter (1997) failed to find gender differences in a sample of marketing managers. Other authors as well were unable to document significant gender differences concerning the impostor phenomenon (e.g., Cozzarelli and Major, 1990; Fried-Buchalter, 1992; Lester and Moderski, 1995; Ferrari and Thompson, 2006; Harvey, 1981, unpublished). On the contrary, in the study by Topping and Kimmel (1985) on a group of university teachers men reported even stronger impostor concerns than women. In summary, men as well appear to be susceptible to impostor feelings, and the question of potential gender effects must be regarded as unresolved.

### Personality Correlates and Independence of the Impostor Phenomenon

Various personality correlates raise the question whether the impostor phenomenon constitutes a construct in its own right or whether it is merely an aspect of some other, more comprehensive personality traits. Clance and Imes (1978) stated already in their first publication that experiencing the impostor phenomenon is accompanied by higher anxiety, low self-esteem, and depressive symptoms: “The clinical symptoms most frequently reported are generalized anxiety, lack of self-confidence, depression, and frustration related to inability to meet self-imposed standards of achievement” (p. 2). This could be confirmed in subsequent research: A study by Chrisman et al. (1995) reveals a moderate to high significant correlation between impostor phenomenon and depression, fear of failure, and fear of negative evaluation (Chrisman et al., 1995). In addition, a study by Thompson et al. (1998) shows that persons with an impostor self-concept reach significantly higher anxiety scores than others. Chrisman et al. (1995) also report significant and highly negative correlations between the impostor phenomenon and self-esteem. Moreover, Ross et al. (2001), who examine associations between impostor phenomenon and the Big Five personality factors in a sample of college students, report high positive associations with neuroticism and significant negative associations with the factors extraversion and conscientiousness which can be regarded as moderate. There are no significant correlations with the factors openness for experience and agreeableness, although Chae et al. (1995) report a low correlation with agreeableness in a similar study. In all, the high positive correlation with neuroticism as well as the moderately negative correlation with extraversion is in accord with conceptions of the impostor phenomenon (Clance and Imes, 1978). Besides, individuals experiencing the impostor phenomenon exhibit characteristics which are likely to be highly correlated with the personality construct of core self-evaluations (CSE; Judge et al., 2003; Heilmann and Jonas, 2010), but in a negative form. Core self-evaluations denote a higher-order construct comprising high self-esteem and self-efficacy, internal locus of control, and low neuroticism. According to pertinent investigations, individuals with impostor self-concept are characterized by low self-esteem; as Clance (1985) states, they fear that they will never be able to reproduce good performances they achieved in the past. This corresponds to the CSE-component low self-efficacy. In addition, they do not attribute achievements to internal factors but to uncontrollable external factors, a fact which speaks against an internal control conviction in these individuals. A further consideration is that the dynamics of narcissism might be involved in the facets of the impostor phenomenon, since individuals may heavily try to live up to an idealized self-image of being intelligent in order to get the validation necessary to feel good about themselves (Langford and Clance, 1993).

Persons with an impostor self-concept on principle doubt themselves and their own competences; they live in fear of failing and of 1 day being exposed as incapable. According to Clance et al. (1995), the apprehensions, self-doubts, and fears connected with this phenomenon emerge especially with new tasks and challenges, so that these persons tend toward two coping strategies to protect their endangered self-concept: On the one hand, they overcompensate their fears by meticulous preparation and extreme effort (over-doing/perfectionism), on the other hand, they engage in self-handicapping behaviors, e.g., postponing work for so long that it can barely be accomplished (under-doing/procrastination).

Perfectionism is defined as a personality disposition characterized by striving for flawlessness and setting exceedingly high standards for performance accompanied by the tendency for overly critical evaluations of one’s behavior (Frost et al., 1990; Flett and Hewitt, 2002). As such, it pervades all areas of life, particularly work and studies (Stoeber and Stoeber, 2009). Recent conceptualizations view the construct as multifaceted in nature (e.g., Frost et al., 1990; Hewitt and Flett, 1991), while these facets have been conceptually and empirically combined to higher order dimensions of perfectionistic concerns (Frost et al., 1993; Stoeber and Otto, 2006). A widely used multidimensional model is that of Frost et al. (1990), which differentiates six dimensions of perfectionism: personal standards, concern over mistakes, parental expectations and criticism, doubts about actions, and organization. Perfectionism is believed to have a marked impact on the emergence and maintenance of impostor feelings (Kets de Vries, 2005; Sakulku and Alexander, 2011). Consistently, a positive relationship between characteristics of impostorism and perfectionism has been supported by a series of empirical studies. Individuals with impostor feelings also tend to experience perfectionistic cognitions (Thompson et al., 1998; Ferrari and Thompson, 2006), and self-evaluative perfectionism (Dudău, 2014). Thompson et al. (2000) found that impostor fears were related to an exaggerated, perfectionistic concern over making mistakes, and a greater tendency to overestimate the number of mistakes they had made than non-impostors. In addition, Ferrari and Thompson (2006) showed in a sample of undergraduate students that impostor fears were moderately correlated with perfectionistic self-presentation, such as perfectionistic thoughts about avoiding imperfection, non-display of imperfection, and the need to appear perfect (cf. Frost et al., 1995). A study by Cromwell et al. (1990) revealed that, compared to non-imposters, persons high in impostorism feel they need to achieve perfection in order to gain others’ approval. Thompson et al. (2000) found that impostors show a higher level of fear of negative evaluation and that the motive behind their achievement behavior is to meet their perception of other peoples’ standards. These perceived social expectations may contribute to perfectionism in impostors.

Procrastination as the “tendency to put off or completely avoid an activity under one’s control” (Tuckman, 1991, p. 474) is a performance-related behavior associated with perfectionism (Shafran and Mansell, 2001; Flett et al., 2004). Previous studies have provided empirical evidence that a substantial positive association exists between impostor phenomenon and self-handicapping behaviors, i.e., procrastination (Ross et al., 2001; Cowman and Ferrari, 2002; Want and Kleitman, 2006). Cowman and Ferrari (2002) found that self-handicapping together with shame-proneness best predicted imposter fears among students. Consistent with this outcome, Ferrari and Thompson (2006) reported that impostor scores by students correlated positively with favorable impression management strategies, and that impostors were more likely than were non-impostors to engage in self-handicapping actions (e.g., not practicing before an upcoming task).

Together these studies suggest that imposters tend to procrastinating behaviors which allow for attributing the envisaged failure to insufficient preparation time instead of having to acknowledge that its cause is a lack of abilities (Cowman and Ferrari, 2002; Want and Kleitman, 2006). The other alternative is the extreme opposite and aims at preventing a potential failure with the help of excessive efforts, so that these individuals work much harder than is actually necessary. Since both of these behavioral tendencies, as well as the impostor-typical achievement-related self-doubts and fears of failure are associated with elevated stress experience (cf. Kammeyer-Mueller et al., 2009), individuals with impostor phenomenon are likely to feel particularly strained by their work.

To clarify the above-mentioned associations, more evidence is needed because the majority of previous studies on the impostor phenomenon relied on student samples rather than samples of professionally successful persons (Kolligian and Sternberg, 1991; Chrisman et al., 1995; King and Cooley, 1995; Henning et al., 1998; Thompson et al., 1998; Leary et al., 2000; Ross et al., 2001; September et al., 2001; Sonnak and Towell, 2001; Bernard et al., 2002; Ross and Krukowski, 2003; Kumar and Jagacinski, 2006; French et al., 2008). Therefore, the current research aims at clarifying the following issues:

(a) As the question of possible gender differences has not been solved (cf. Kaplan, 2009), we examine whether the impostor phenomenon is a gender-typical phenomenon. In accord with recent studies, we expect no gender differences.
(b) As for potential effects of the impostor phenomenon on working styles it is conceivable that aﬄicted individuals either seek to exclude mistakes and reduce the risk of failure with the help of very thorough and extensive preparatory work (e.g., Thompson et al., 1998, 2000; Ferrari and Thompson, 2006) or that they habitually use self-handicapping behaviors, i.e., procrastination, in an attempt to reduce the significance of possible failures or negative evaluations (e.g., Ross et al., 2001; Cowman and Ferrari, 2002). Therefore, we expect that the impostor phenomenon is associated with perfectionist and with procrastinating behaviors (hypothesis 1).
(c) Since these dispositional associations outlined above can be assumed to have stress-enhancing effects (cf. Kammeyer-Mueller et al., 2009), we assume that the impostor phenomenon is related to higher levels of strain experience (hypothesis 2).
(d) Earlier research has revealed that the impostor phenomenon is associated with a number of other constructs (e.g., Chrisman et al., 1995; Thompson et al., 1998; Ross et al., 2001). We expect to replicate the positive correlations with neuroticism, dysthmia, and anxiety, as well as negative correlations with euthymia and conscientiousness. Based on theoretical considerations, indicators of positive self-evaluations are expected to negatively correlate with the impostor phenomenon; narcissism is expected to positively correlate with the impostor phenomenon (hypothesis 3).
(e) Nevertheless, the impostor phenomenon can be assumed to possess discriminant validity. We hypothesize that the impostor phenomenon can be distinguished from these constructs as a construct in its own right (hypothesis 4).

## Materials and Methods

### Sample

A total of 457 persons opened the link to the online study. Two hundred and fifty two participants engaged in the survey. Ten individuals reported to possess neither leadership experience nor employee responsibility, and therefore they were excluded from the study sample. The remaining 242 participants (37% women) occupied leadership positions in various sectors. Their ages ranged from 24 to 67 years (*M* = 44.3 ± 9.02) and they had on average 10.73 years of experience in leadership positions. Of these 242 people, 190 (78.5%) filled in the complete questionnaire. Neither was there a difference in gender, *F*(1,240) = 1.58, *p* = 0.21, nor educational level, *F*(7,242) = 12.25, *p* = 0.09 between those who finished the questionnaire and those who dropped out.

Potential participants were directly contacted via e-mail. To this end, we used private as well as professional contacts, approached institutions associated and cooperating with Frankfurt University (e.g., Scientific Society) and the university’s alumni network. Moreover, potential participants were contacted via the mailing list of a network of women in leadership positions and a network of leaders in the financing and consulting sector. The cover letter included some information as well as a direct link to the survey. In addition, all contacted persons were asked to forward the request to acquaintances in leadership positions, in order to reach as many leaders as possible via “snowballing.” Participants were given the opportunity to receive feedback on their personality profile at the end of the study.

### Ethics Statement

Ethical approval was granted by the author’s institutional ethics committee (Goethe University Frankfurt, Department of Psychology and Sports Ethics Committee). The surveys did not collect any identifying or health information from the participants. The introduction page clearly stated that participation was voluntary and anonymous, and that participants had the right to withdraw from the study at any time. The authors had no access to any participant information, because data collection was anonymous.

The study was conducted in agreement with the Declaration of Helsinki and with the “Common Rule” of the US Department of Health and Human Services.

### Procedure

Having received the link to the survey, the participants could answer the online questionnaire at any time using any computer with access to the internet. All participants received the online questionnaire in the same order. The instructions were standardized for the entire sample and thus warranted objectivity of the procedure. The study was presented under the title “Self-concept and decision behavior.” Completing the survey took 25 min on average.

### Instruments

The questionnaire set included the instruments mentioned below. Except for the State-Trait-Anxiety-Depression-Inventory (STADI; Laux et al., 2013), all responses were marked on 6-point scales (1 = not at all apply, 6 = totally apply), to keep the task as simple as possible.

#### Big Five

To assess the Big Five personality traits, we used the Big Five Inventory by Rammstedt and John (2005) in its short version (BFI-K). In the BFI-K, four items each measure the scales of extraversion (e.g., “I am rather reticent, standoffish”), agreeableness (e.g., “I tend to criticize others”), conscientiousness (e.g., “I complete tasks thoroughly”) and neuroticism (e.g., “I get easily depressed, gloomy”); five items represent the subscale openness for experiences (“I am interested in many things”). Internal consistencies were α = 0.74 (extraversion), α = 0.66 (agreeableness and conscientiousness), α = 0.74 (neuroticism) and α = 0.72 (openness).

#### Impostor Phenomenon

The impostor phenomenon was assessed with the 20-item Clance Impostor Phenomenon Scale (CIPS; Clance, 1985). An example item is: “I can give the impression that I am more competent than I really am.” The scale had an internal consistency of α = 0.92.

#### Core Self-Evaluations

The Core Self-Evaluations Scale (CSES) developed by Judge et al. (2003) was used in its German translation (Heilmann and Jonas, 2010) to measure an individual’s fundamental attitude toward herself or himself in the present study. The CSES consists of 12 items (e.g., “I doubt my competence”) and measures the four traits self-esteem, self-efficacy, internal control conviction, and emotional stability. Internal consistency of the scale was α = 0.86.

#### Anxiety and Depression

Anxiety and depression were assessed with the State-Trait-Anxiety-Depression-Inventory (STADI; Laux et al., 2013). Participants of the present study were given only the 20 items measuring habitual anxiety and depression (trait items), with a 4-point answering scale (1 = almost never, 4 = almost always). Anxiety was measured with the two subscales agitation (five items, e.g., “I am easily tense”) and apprehension (five items, e.g., “I worry about problems that might occur”). Both scales have an internal consistency of α = 82. Depression was assessed with the two scales dysthymia (five items; e.g., “I am despondent”) and euthymia (five items; e.g., “I enjoy life”). Internal consistencies were α = 0.80 (dysthymia) and α = 0.90 (euthymia).

#### Perfectionism

To measure perfectionism we used 17 items from the Frost Multidimensional Perfectionism Scale (FMPS, Frost et al., 1990) in the German translation by Stoeber (1995a, unpublished). This scale is originally comprised of 35 items, which, according to Stoeber (1998), can be assigned to four subscales: Concern over Mistakes and Doubts, Personal Standards, Parental Expectations and Criticism, and Organization. Given the time-related constraints of an online-study, there was a need to select scales directly of interest to our research question. Based on the psychometric parameters reported in the literature as well as content-related aspects, we used the subscales “Personal Standards” (e.g., “I expect myself to show higher performance in my daily tasks than most other people”; α = 0.82) and “Concern over Mistakes and Doubts (e.g., “If I fail at work, I should fail as a person”) for assessing perfectionism. Both of these facets of perfectionism are likely to substantially influence task-related behaviors. Using the Spearman–Brown formula, the latter subscale was shortened to 10 items (FMPS: 9, 14, 17, 18, 21, 23, 25, 28, 32, and 34); internal consistency was good (α = 0.85). We did not assess the subscales “Parental Expectations and Criticism,” because our focus was on the working styles associated with impostorism. The “Organization” subscale, however, was not considered in our analyses because it is only loosely related to the other FMPS subscales and is excluded when calculating its total score (cf. Frost et al., 1990).

#### Procrastination

To assess procrastination we used the nine items with the highest item-part whole correlations from the Tuckman Procrastination Scale (Tuckman, 1991) in the German translation by Stoeber (1995b, unpublished; TPS-D; e.g., “When I have a deadline I wait until the last minute,” included items: 1, 3, 4, 11, 12, 13, 14, 15, and 16). Internal consistency of the scale was α = 0.92.

#### Narcissism

To assess narcissism we presented seven items from the Narcissistic Personality Inventory (NPI) by Raskin and Terry (1988) in the German version by Stucke (2000; e.g., “I like to be the center of attention”). The short version of the NPI was also reduced to seven items, namely those which showed the highest discriminatory power in earlier studies (Schütz et al., 2004, included items of the NPI: 7, 9, 10, 12, 13, 30, and 36). Internal consistency was α = 0.83.

#### Strain

We assessed level of strain with the Irritation Scale by Mohr et al. (2007). Irritation is defined as subjectively perceived emotional and cognitive strain in occupational contexts. The scale comprises a total of eight items (e.g., “After work it is difficult for me to unwind”); internal consistency was α = 0.88.

Apart from these instruments we assessed the demographic variables age, gender, and duration of leadership experience.

## Results

### Impostor Phenomenon – Association with Gender, Other Trait Variables, and Strain

Correlations are displayed in **Table 1**. There is almost no association between the impostor phenomenon and gender. To control for alpha inflation when interpreting the bivariate correlations of impostor phenomenon with trait variables and strain, we applied the Bonferroni–Holm method (Holm, 1979). Below, we comment on the significant correlations.

**Table 1:** Descriptive statistics and intercorrelations of all variables.

		*M*	*SD*	1	2	3	4	5	6	7	8	9	10	11	12	13
(1)	Gender^a^	0.63	0.48													
(2)	Impostor phenomenon	2.48	0.81	-0.08												
(3)	Neuroticism	2.66	0.90	-0.13	0.48**											
(4)	Agitation	1.94	0.60	-0.03	0.37**	0.69**										
(5)	Apprehensiveness	1.74	0.55	0.06	0.48**	0.68**	0.59**									
(6)	Dysthymia	1.51	0.47	-0.07	0.47**	0.63**	0.45**	0.53**								
(7)	Euthymia	2.95	0.68	-0.06	-0.15*	-0.47**	-0.06	-0.16*	-0.31**							
(8)	Core self-evaluations^b^	4.57	0.70	0.04	-0.46**	-0.65**	-0.22**	-0.40**	-0.53**	0.61**						
(9)	Narcissism	3.55	0.89	-0.04	-0.15*	-0.22**	-0.03	-0.07	-0.10	0.33**	0.38**					
(10)	Personal Standards^c^	3.49	0.97	-0.01	0.21**	0.15	0.27**	0.12	0.09	0.08	0.02	0.37**				
(11)	Concern over Mistakes and Doubts ^c^	2.19	0.76	-0.07	0.57**	0.49**	0.46**	0.41**	0.37**	-0.27**	-0.49**	0.07	0.52**		.	
(12)	Procrastination	2.71	0.97	-0.08	0.36**	0.30**	0.17*	0.24**	0.34**	-0.15*	-0.45**	-0.06	0.08	0.36**		
(13)	Conscientiousness	4.51	0.77	-0.17	-0.19*	-0.19**	0.00	-0.09	-0.18*	0.27**	0.49**	0.32**	0.30**	-0.17	-0.49**	
(14)	Strain	2.64	0.92	-0.06	0.42**	0.65**	0.67**	0.53**	0.47**	-0.24**	-0.28**	<0.00	0.35**	0.47**	0.34**	-0.06

N = 183-242 (differences in N are due to pairwise deletion because of incomplete item responses in personality questionnaires). ^a^0 = female; 1 = male. ^b^multidimensional construct comprising self-esteem, self-efficacy, internal locus of control and emotional stability, ^c^component of perfectionism; *p < 0.05, **p < 0.01.

There is a strong and positive correlation with dimensions of perfectionism as expected in hypothesis 1, but the strong association is restricted to Concern over Mistakes and Doubts (*r* = 0.57), while the association with personal standards is weak (*r* = 0.21). Thus, leaders with an impostor self-concept report being afraid to lose the respect and affection of their social environment in the case of failure.

As also expected in hypothesis 1, there is also a moderate association with procrastination (*r* = 0.36).

Correspondingly, the impostor phenomenon is highly positively correlated with the subjective experience of work-related stress and strain (*r* = 0.42), as expected in hypothesis 2.

Furthermore, there is a strongly positive correlation between the impostor phenomenon and neuroticism (*r* = 0.48) and dysphoric mood (*r* = 0.47). In addition there is a strongly positive correlation between the impostor phenomenon and anxiety, the association with general apprehension was stronger than with agitation [*r*_ap_ = 0.48; *r*_ag_ = 0.37; *t*(204) = 1.98^2^, *p* = 0.049, two-sided]. Moreover, the impostor phenomenon shows high negative correlations with central indicators of positive self-evaluation (core self-evaluations; *r* = -0.46) such as self-esteem, self-efficacy, internal control conviction, and emotional stability. There is also a negative but weak correlation with conscientiousness (*r* = -0.19). The negative correlation with euthymia (*r* = -0.15), i.e., the experiencing of positive emotions, and narcissism are not significant when controlling for alpha inflation. These results are consistent with the correlations between the impostor phenomenon and personality traits predicted by hypothesis 3.

### The “Impostor Phenomenon” as an Independent Construct

Given the strong empirical overlap of the impostor phenomenon with the traits neuroticism, depression, anxiety (apprehension), core self-evaluations, and perfectionism (Concern over Mistakes and Doubts), the discriminant validity of impostorism versus these five scales was examined with the help of confirmatory factor analyses (CFA).

To reduce the number of variables, we randomly split the 20 items of the CIPS, the twelve items of the Core Self-Evaluations scale, and the ten items of the subscale “Concern over Mistakes and Doubts” into two item sets, respectively, so-called item parcels (cf. Marsh et al., 1998). In the following CFAs the item parcels of the said scales were used as indicator variables of the constructs.

**FIGURE 1:**
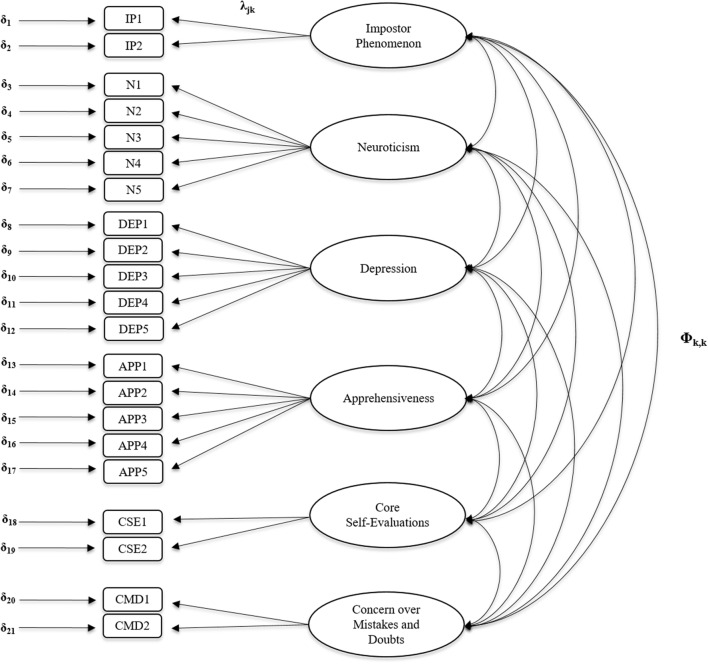
**Six-factor model.** Ellipses describe latent factors, rectangles manifest indicator variables; j, manifest variable; k, latent variable; δj, error of the j-th manifest indicator variable; λjk, factor loadings; Φk,k, correlations of the latent variable; IP, impostor phenomenon; N, neuroticism; DEP, depression; APP, apprehension (anxiety); CSE, core self-evaluations; CMD, concern over mistakes and doubts.

To validate the construct impostor phenomenon we then calculated CFAs for five competing models. We assumed an unrestricted model where all six constructs constitute independent, but correlated factors; this model was compared to five restricted models. The initial model is depicted in **Figure 1**.

The reduced models were created by combining the impostor phenomenon and one of the five other constructs into a joint factor. Consequently, these models contained five factors each: the joint factor consisting of the impostor phenomenon, and one of the other five factors plus the four remaining factors. The purpose of the CFA was to test whether the unrestricted model exhibits a better model quality and generally fits better than one of the restricted models. If the results show that the impostor phenomenon constitutes a factor in its own right, this confirms the validity of the construct. To assess model quality, the following fit indices were calculated: (a) Root Mean Square Error of Approximation (RMSEA; Steiger, 1990), (b) Standardized Root Mean Error Residual (SRMR; Bentler, 1995), (c) Comparative Fit Index (CFI; Bentler, 1990), and (d) Tucker Lewis Index (TLI; Tucker and Lewis, 1973). In interpreting model fit we relied primarily on these indices of practical fit rather than the chi-square index, because chi-square is extremely sensitive to sample size. Also, the models are not nested models, such that the chi-square difference test is not appropriate. Instead, we considered differences in the Aikaike information criterion (AIC). Correlations between factors were permitted in all models. Model fit indices are reported in **Table 2**.

**Table 2:** Fit indices of the unrestricted and restricted models.

Model fit index	Unrestricted 6-factor model (Impostorism as construct of its own)	Restricted 5-factor model A (Impostorism = Concern over mistakes)	Restricted 5-factor model B (Impostorism = Neuroticism)	Restricted 5-factor model C (Impostorism = Depression)	Restricted 5-factor model D (Impostorism = Apprehension)	Restricted 5-factor model E (Impostorism = Core self-evaluations)
RMSEA	0.053	0.064	0.085	0.092	0.090	0.071
SRMR	0.060	0.064	0.070	0.070	0.072	0.084
CFI	0.948	0.921	0.863	0.837	0.846	0.903
TLI	0.937	0.907	0.835	0.806	0.817	0.885
AIC	6965.052	7007.188	7111.908	7155.908	7140.281	7039.205

*Model A, common latent factor for impostor phenomenon and concern over mistakes; Model B, common latent factor for impostor phenomenon and neuroticism; Model C, common latent factor for impostor phenomenon and depression; Model D, common latent factor for impostor phenomenon and apprehension; Model E, common latent factor for impostor phenomenon and core self-evaluations; RSMEA, Root Mean Square Error of Approximation; SRMR, Standardized Root Mean Error Residual; CFI, Comparative Fit Index; TLI, Tucker Lewis Index; AIC, Aikaike Information Criterion.*

Overall, the unrestricted model fitted the empirical data reasonably well. RMSEA (0.053) and SRMR (0.060) suggested good fit, which was also true for the CFI (0.948) and TLI (0.937).

Subsequently five restricted models were created which contain four factors each (models A–E). In model A, the constructs neuroticism, depression, apprehension, and core self-evaluations are independent factors, whereas the impostor phenomenon and concern over mistakes and doubts form a joint factor. As to be seen from **Table 2**, this restricted model fitted the data worse. In the second restricted model (B), the constructs impostor phenomenon and neuroticism were combined to form a joint factor, while the other three constructs function as independent factors. Again, this model fitted the data worse than the unrestricted model. Similar results derived from the analysis of model C (combining impostor phenomenon and depression), model D (combining impostor phenomenon and apprehension), and model E (combining impostor phenomenon and core self-evaluations). Therefore, the unrestricted model including the impostor phenomenon as separate dimension was more appropriate than any of the restricted models. In all, results of the CFAs indicated that the impostor phenomenon is a construct in its own right and can be distinguished from the constructs of neuroticism, depression, apprehension, core self-evaluations, and perfectionism. As expected in hypothesis 5, the imposter phenomenon is associated with a number of other constructs, but it can be distinguished from these as a construct in its own right. Hence, discriminant validity can be assumed for this trait; this can be interpreted as construct validity.

## Discussion

### Impostor Phenomenon – Gender

In our study we found no association between impostor phenomenon and gender, which is in accord with findings of various other investigations (e.g., King and Cooley, 1995; Harvey, 1981, unpublished). While contradicting the initial assumptions of Clance and Imes, this finding largely confirms the results of earlier studies which also investigated successful professionals (such as managers; Fried-Buchalter, 1997). Some studies (e.g., King and Cooley, 1995) based on student samples, however, showed that female students experienced the impostor phenomenon more frequently and more intensively than male students. The question arises whether the different types of samples can explain the contradictory findings. A possible explanation would be that although the impostor phenomenon is generally more frequent and more pronounced in women, this does not concern those women who manage to advance into positions that are generally difficult to attain, particularly for women. This assumption is based on the idea that women in leadership positions possess certain characteristics which are generally attributed to men, such as goal orientation, self-confidence, and assertiveness, or that they develop these characteristics in order to meet the challenges of male-dominated professional domains (cf. Wolf, 2002). Female authority figures would probably not be successful if they behaved in accord with the feminine gender stereotype (cf. Wolf, 2002). Various investigations show that female leaders differ more from other women than from their male colleagues. This is attributed to higher levels of dominance, success potential, self-affirmation, commitment, or work orientation (e.g., Weinert, 1990; Rastetter, 2007). In principle, it is conceivable that so-called “career women” either develop such behaviors over the course of their career or that there is some sort of selection effect, in that primarily such women gain access to leadership roles who possess these qualities in the first place. A similar principle can be assumed to hold for the impostor phenomenon: It is possible that the feelings connected with the phenomenon decrease in the course of professional advancement - and in the course of developing the said characteristics - or that women in leading positions are generally less prone to experience the impostor phenomenon than other women.

### Impostor Phenomenon and Personality Correlates

In line with studies from the US (e.g., Chrisman et al., 1995; Ross et al., 2001), we were able to confirm the expected correlations between impostor phenomenon and the assessed characteristics: The impostor phenomenon is accompanied by higher levels of anxiety and dysphoric mood as well as emotional instability and an overall negative self-evaluation. In this context, anxiety is characterized more by general apprehension than by tenseness. Persons experiencing the impostor phenomenon thus seem to be affected primarily by cognitive components of anxiety (e.g., doubts, apprehension, and lack of confidence) rather than by emotional or physical tenseness (such as physical restlessness, nervousness). Similarly, with respect to the trait depression, there are high positive associations with dysphoric moods, but only weak negative ones with euthymia. Individuals with impostor self-concept are thus less characterized by the inability of experiencing positive emotions, such as joy, than by the inability to control their fears and apprehensions. The present findings suggest that the main problematic of the impostor phenomenon is of a cognitive rather than an emotional nature, which corresponds to the experience of anxiety described above. Considering that individuals experiencing the impostor phenomenon report fears of failure and the fear of one day being exposed as a phony, it can be assumed that these individuals are generally particularly anxious. Due to their failure to internalize success it is probable that they also have low self-esteem and a tendency toward dysphoric moods.

As for the working styles associated with the impostor phenomenon, we found that persons with an impostor self-concept tend to show both perfectionist and procrastinating behaviors as expected in hypothesis 1. Frost et al. (1995) stated that the difference between the impostor phenomenon and perfectionism is that perfectionists will not disclose their mistakes to others, fearing to be viewed as imperfect. Imposters, however, openly communicate their self-perception of imperfect performance to others (Ferrari and Thompson, 2006). Our data show that leaders with an impostor self-concept report being afraid to lose the respect and affection of their social environment in the case of failure. To understand the strong associations between both constructs, perfectionism may be regarded as a predisposing and maintaining factor of impostorism (Kets de Vries, 2005; Sakulku and Alexander, 2011).

At first glance, it may seem contradictory to state that individuals are both perfectionist, setting high standards for themselves and their performance, and that they also display the tendency to postpone pending professional tasks. An explanation for the coexistence of both working styles might be that persons with an impostor self-concept generally tend toward perfectionism, but use procrastinating behaviors to protect their already weakened self-concept – in the sense of “if I don’t try it, I cannot fail” (see also Cowman and Ferrari, 2002; Ferrari and Thompson, 2006). Thus, some persons in leadership positions experiencing the impostor phenomenon have difficulties in disciplining themselves and in tackling important tasks. Instead they tend to postpone tasks even though they are aware that this behavior is dysfunctional. As a consequence, they often experience time pressure. This assumption is supported by empirical studies which report strong associations between impostor phenomenon and *self-handicapping* strategies (e.g., Ross et al., 2001; Want and Kleitman, 2006).

Another explanation for the coexistence of perfectionism and procrastination could be that persons with an impostor self-concept, though inclined to postpone professional tasks and duties, exhibit perfectionist working styles such as overly thorough preparation or generally very intensive work to impress their environment with such behaviors (cf. Ferrari, 1992). This is supported by the assumption of Langford and Clance (1993) that persons experiencing the impostor phenomenon are particularly concerned with making a positive impression on others.

The protective effect of these strategies appears, however, to be highly limited: In case of success, the individuals concerned do not attribute their achievement – or their avoidance of failure – to their own abilities, but, after perfectionist preparation, to their excessively hard work or, after procrastination, to external factors, such as luck or favorable circumstances. As a consequence, the elation and relief provided by the achievement is only of a short duration. Due to the lack of internalization, the new achievement rapidly intensifies the feeling of being a fraud, rather than resulting in increased self-confidence. This, in turn, increases the fear of not being able to repeat the achievement in the future, so that the experienced fears, apprehensions and self-doubts reappear with every new task. This causes a circle of over-meticulous preparation/postponing, short-term relief, and self-doubt, which leads to the persistence or even future intensification of impostor feelings, for it can be expected that demands will grow with every further achievement of the individual in question.

As could be expected, our study shows that the impostor phenomenon is accompanied by an increased level of work-related strain as expected in hypothesis 2. In this context, it can be assumed that not only the dysfunctional working styles enhance stress, but that other aspects such as emotional instability (neuroticism) or negative self-evaluation (e.g., Kammeyer-Mueller et al., 2009) as well evoke increased stress levels in the persons concerned. This raises the question which long-term effects can be reckoned with in persons with an impostor self-concept.

### Impostor Phenomenon and Independence of the Construct

In the framework of construct validation we investigated whether and to what extent the impostor phenomenon can be distinguished from other, already established constructs, i.e., we tested the discriminant validity of the construct. The reason for doing so was that there are high correlations between impostor phenomenon and the constructs of depression, neuroticism, anxiousness (apprehension), core self-evaluations, and perfectionism (i.e., Concern over Mistakes and Doubts). The initial model, which treats the impostor phenomenon as a factor of its own, exhibits a good to acceptable model fit and can be differentiated from other, restricted models, i.e., from the constructs mentioned. Importantly and in contrast to previous research, this is one of the few studies which analyzed the impostor phenomenon among a non-student sample. The results confirm the assumption that the impostor phenomenon is indeed prevalent among people with successful careers and forms a construct in its own right as expected in hypothesis 4.

## Conclusion

To sum up, the present study contributes to validating the construct impostor phenomenon. Based on correlations, we were able to replicate earlier findings considering the validity of the impostor phenomenon. Yet, the present study demonstrates this effect in a sample of individuals occupying leadership positions, which emphasizes the external validity of our findings.

Although the phenomenon correlates with higher levels of anxiety, dysphoric mood, emotional instability, an overall negative core self-evaluation as well as perfectionism (i.e., concern over mistakes and doubts), as already predicted by hypothesis 4, the construct could be distinguished from related constructs with the help of CFA and can thus be considered an independent construct.

In all, the findings indicate that the impostor phenomenon is a dysfunctional personality style. Persons with an impostor self-concept tend toward both perfectionist and procrastinating behaviors and also report higher work-related strain. Although the impostor phenomenon neither reflects a psychological disorder requiring treatment nor hinders the attainment of success (Ross and Krukowski, 2003), it negatively affects well-being because it prevents people from enjoying their merits (Clance and O’Toole, 1988). The impostor phenomenon negatively relates to conscientiousness and achievement-striving (Chae et al., 1995; Ross et al., 2001; Bernard et al., 2002; Want and Kleitman, 2006), which is indicative of impostors’ inclination toward procrastination and self-handicapping. Therefore, the construct seems to be highly relevant in the context of human resources development, coaching and training as well as from the perspective of psychological treatment. The aim would be to improve self-esteem and self-evaluation as well as to teach new coping strategies to achieve a general stress reduction. In this context, competence trainings and cognitive restructuring of dysfunctional cognitions/concepts could be used in the framework of behavioral therapy.

## Author Contributions

SR substantially contributed to the conception and design of the work, interpreted the data, wrote the manuscript, and revised it critically for important intellectual content. MB supervised the design of the study and data collection and contributed to data analysis as well as writing and revising the manuscript. ML contributed to designing and conducting the study, analyzing the data as well as writing the manuscript. All authors contributed to and approved this final version of the manuscript.

## Conflict of Interest Statement

The authors declare that the research was conducted in the absence of any commercial or financial relationships that could be construed as a potential conflict of interest.

## Footnotes

1 In this paper, the terms impostor phenomenon, imposter self-concept and impostorism are used interchangeably.

2 The *t*-statistic provided here refers to Williams (1959).
